# 2591. Anxiety and depression among patients with non-tuberculous mycobacterial disease in Shanghai: A cross-sectional study

**DOI:** 10.1093/ofid/ofad500.2206

**Published:** 2023-11-27

**Authors:** zheng yuan, Bijie Hu, sikang ni

**Affiliations:** Zhongshan Hospital, Fudan University, shanghai, Shanghai, China; Department of Infectious Diseases, Zhongshan Hospital, Fudan University, Shanghai, Shanghai, China; Zhongshan Hospital, Fudan University, shanghai, Shanghai, China

## Abstract

**Background:**

To understand the mental health status and its influencing factors among patients with non-tuberculous mycobacterial disease and to provide a reference for medical staff to formulate scientific and feasible intervention strategies.

**Methods:**

A total of 114 patients diagnosed with non-tuberculous mycobacillosis during hospitalization in the Department of Infection from September 2020 to April 2021 were selected as the research participants. Participants’ mental health status and related factors were evaluated using a self-made general patient information questionnaire, self-rating Anxiety Scale (SAS), and self-rating Depression Scale (SDS).

**Results:**

Among 114 patients with non-tuberculous mycosis, 61 (53.51%) exhibited depressive symptoms, and the SDS score was 51.15 ± 13.04, which was higher than the national norm of 41.88 ± 10.57 (P < 0.05); further, 39 patients (34.21%) showed anxiety symptoms, and the SAS score was 45.75 ± 10.81, which was significantly higher than the national norm of 29.78 ± 10.07 (P < 0.05). Body mass index and monthly household income had significant effects on depression in patients with non-tuberculous mycobacterial disease (P < 0.05). Educational level had a significant effect on the anxiety state of patients with non-tuberculous mycobacterial disease (P < 0.05).

**Conclusion:**

Patients with non-tuberculous mycobacterial disease are prone to depression and anxiety. Nurses should pay attention to it in clinical work for the timely identification of and intervention for anxiety and depression and intervene.

**Disclosures:**

**All Authors**: No reported disclosures

